# Multi-scale Gaussian representation and outline-learning based cell image segmentation

**DOI:** 10.1186/1471-2105-14-S10-S6

**Published:** 2013-08-12

**Authors:** Muhammad Farhan, Pekka Ruusuvuori, Mario Emmenlauer, Pauli Rämö, Christoph Dehio, Olli Yli-Harja

**Affiliations:** 1 Department of Signal Processing, Tampere University of Technology, 33720 Tampere, Finland; 2 Biozentrum, Universität Basel, 4056 Basel, Switzerland

## Abstract

**Background:**

High-throughput genome-wide screening to study gene-specific functions, e.g. for drug discovery, demands fast automated image analysis methods to assist in unraveling the full potential of such studies. Image segmentation is typically at the forefront of such analysis as the performance of the subsequent steps, for example, cell classification, cell tracking etc., often relies on the results of segmentation.

**Methods:**

We present a cell cytoplasm segmentation framework which first separates cell cytoplasm from image background using novel approach of image enhancement and coefficient of variation of multi-scale Gaussian scale-space representation. A novel outline-learning based classification method is developed using regularized logistic regression with embedded feature selection which classifies image pixels as outline/non-outline to give cytoplasm outlines. Refinement of the detected outlines to separate cells from each other is performed in a post-processing step where the nuclei segmentation is used as contextual information.

**Results and conclusions:**

We evaluate the proposed segmentation methodology using two challenging test cases, presenting images with completely different characteristics, with cells of varying size, shape, texture and degrees of overlap. The feature selection and classification framework for outline detection produces very simple sparse models which use only a small subset of the large, generic feature set, that is, only 7 and 5 features for the two cases. Quantitative comparison of the results for the two test cases against state-of-the-art methods show that our methodology outperforms them with an increase of 4-9% in segmentation accuracy with maximum accuracy of 93%. Finally, the results obtained for diverse datasets demonstrate that our framework not only produces accurate segmentation but also generalizes well to different segmentation tasks.

## Introduction

High-throughput screening used in drug design involves identification of genes which modulate a particular biomolecular pathway. RNA interference (RNAi), by decreasing the expression of particular genes in a cell culture, helps in identifying and analyzing the target gene functions in the cells by observing the cell behavior after gene knockdown [1-3]. Image analysis is at the center stage of such studies where cell cultures are imaged with automated fluorescent microscopy to study the cell behavior in knockdown as well as in normal condition. Genome-wide high-content siRNA screening involves studying the dynamics of gene expression in cellular functions for the whole genome and therefore yields hundreds of thousands of images making their manual analysis impractical [3]. Quantitative image analysis is needed for the identification, classification and quantification of the phenotypes which is also not possible through manual analysis [3,4]. Consequently, fast enough automated image analysis methods are needed to fulfill the potential of high-throughput system.

Segmentation of cells is typically at the core of the image analysis pipelines dealing with high-content genome-wide screening experiments [4,5]. This is generally the step which performs cell detection and further analysis, such as cell tracking and lineage reconstruction and cell classification, is based on the results of cell detection. However, in such experiments, segmentation is challenging due to presence of large number of phenotypes. Different cell phenotypes have different characteristics and appearances and, for some complex and heterogeneous cell cultures, it is difficult to build analysis capable of detecting all the phenotypes, potentially leading to the loss of some phenotypes. Accurate cell segmentation and detection is therefore essential for quantification of phenotypes.

One of the main challenges in cell segmentation is the cells touching and clustering together, forming a clump. Not only the cytoplasms form clumps but clustering of nuclei is also quite common. The latter problem has been tackled in our recent article [6]. The problem with cytoplasm region in general, and specifically with their clumps, is that they do not often have visible boundaries. Due to this reason, and also due to their irregular shapes, the methods typically in use for clump splitting often fail [7]. The other challenge often faced in cytoplasm segmentation is uneven and varying actin signal. Imaging aberrations cause actin signal to be saturated at some locations and to be too low on other locations for being regarded as part of the cell. This causes methods based on global image segmentation methods to fail. Another similar challenge that lies in cytoplasm segmentation is that the inside of the cells is inhomogeneous, consequently the intensity variations are large. Sometimes, part of the cell cytoplasm resembles the background and the methods solely based on image intensity are often found struggling in such situations [4]. However, if along with image intensity, other features locale to those regions are examined, the difference between background and cytoplasm could be highlighted. In addition to all this, uneven illumination and out of focus regions of the image also cause problems in getting accurate segmentation results.

Methods for cell cytoplasm segmentation available in literature can be mainly divided into two approaches: classic segmentation methods and deformable model-based methods. The former includes watershed transform, region growing, and mathematical morphology methods etc., see for example [8,9], whereas the latter comprises active contour [10], level set [11,12] and graph cut based methods [5]. Authors in [7] developed a method in which watershed algorithm with double thresholds is followed by splitting and merging of cellular regions based on quality metric obtained by correctly classified cells. Classification of cells is performed using a set of features with *a priori *information about the cells. In [13], enhancement of high intensity variations in the actin channel is performed by variance filtering. The enhanced image is then smoothed and thresholded using Otsu thresholding method. Subsequently, seeded watershed transform is applied which is restricted to the binary image of the cytoplasm. In another method [5], region growing algorithm and modified Otsu thresholding are used to extract the cytoplasm. Long and thin protrusions on spiky cells are extracted by scale-adaptive steerable filter. Finally, constraint factor graph cut-based active contour method and morphological algorithms are combined to separate tightly clustered cells.

In a method described in [4], the interaction between cells is modeled using a combination of both gradient and region information. Energy function is formulated based on an interaction model for segmenting tightly clustered cells. The energy function is then minimized using a multiphase level set method. Markov Random Fields (MRF) based graphical segmentation model yielding energy minimization problem is also applied to cell cytoplasm segmentation where graph cut method is used to obtain an exact MAP solution [14]. Similarly $P^{n}$ Potts model, where functions of higher-order cliques of pixels are included into the traditional Potts model, combined with learning methods for defining the potential functions accounting for local texture information are used to segment live cell images in [15].

The problem with these methods is that they tend to produce over- and/or under-segmentation, for example, classic segmentation methods. Also, they are sometimes computationally-intensive and slow or they depend on schemes which require parameter initialization, and finding a good set of initial parameters for large heterogeneous dataset often requires user intervention which hinders development of automated analysis pipelines [16]. Moreover, when the cells are non-convex, as in our case, the methods available for segmentation of convex objects do not work, nor do the methods which are based on shape priors.

When cells clump together the cytoplasm outlines become invisible, however the intensity and other features along that part of the image are quite similar to the features of other cell outlines that are visible. Therefore, a segmentation methodology can be developed in which the outlines of the cell cytoplasm are learned by a supervised machine learning algorithm. There are methods in literature [17-20] which use the technique of learning edges for segmentation and object detection. However, all of them detect and model outlines which are distinct, where the outlines are basically used to detect objects or regions in the image utilizing shape information wherever available. In contrast, we need an outline detection technique which not only detects distinct outlines but is also capable of revealing outlines to separate objects of unknown shapes from each other.

In this paper we propose a supervised learning and classification-based cell cytoplasm segmentation methodology in which the outlines of the cell cytoplasm are learnt and detected. A multi-scale approach is used to get the cytoplasm/background segmentation and the detected outlines are overlaid to get the complete segmentation. The results from the classification framework are fed to post-processing phase, where the methodology uses the nuclei segmentation [6] as contextual information to refine the segmentation results.

The rest of the paper is organized as follows: In the Methods section, we describe the proposed cell cytoplasm segmentation methodology. The obtained results are presented and discussed in Results and discussion section. The last section concludes the paper.

## Methods

**Figure 1:**
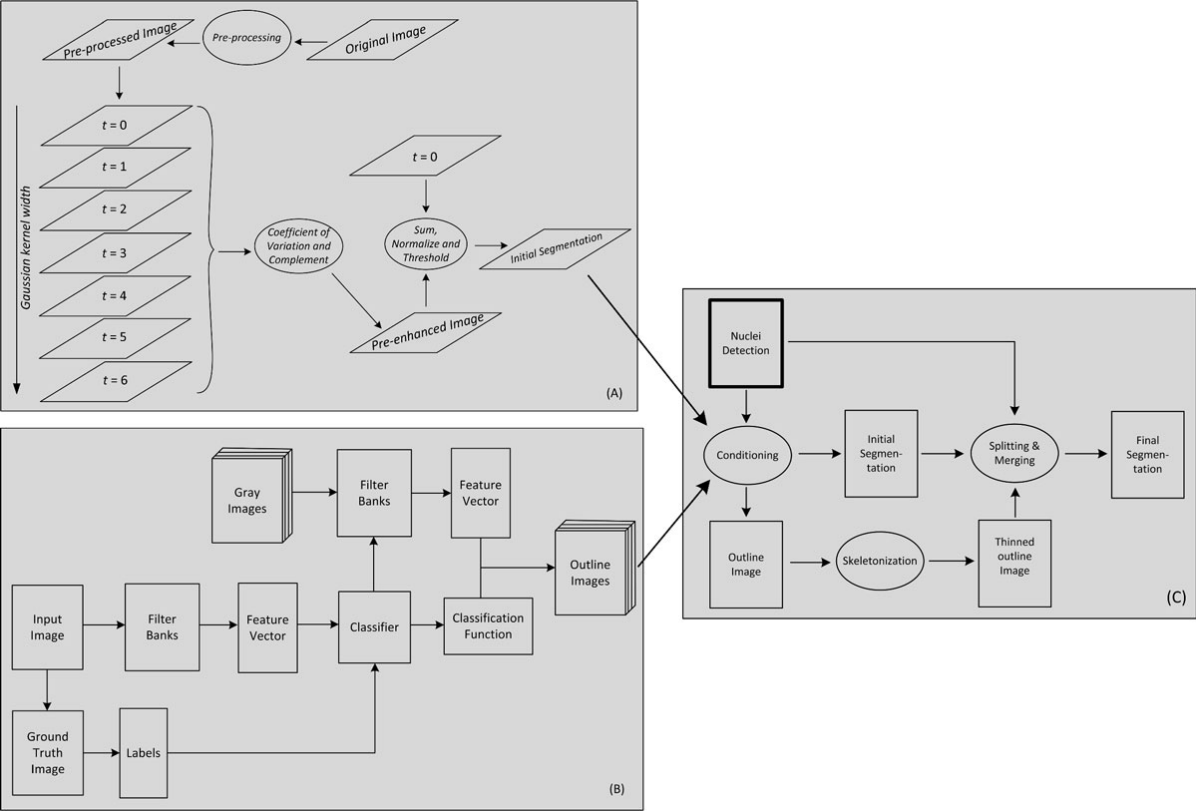
**Block diagram of cytoplasm segmentation methodology**. A block diagram showing the steps performed by the proposed cytoplasm segmentation methodology.

The proposed cell segmentation methodology involves three steps which are delineated by the block diagram in Figure 1. Firstly, images are passed through a pre-processing stage where most of the imaging aberrations are dealt with before applying multi-scale approach to separate cytoplasmic regions from the image background. Secondly, features are extracted from image pixels and a classifier is trained for classification of image pixels as either outline or non-outline to detect the cell outlines. Finally, a post-processing step is performed to refine the outlines so that they form a closed contour around each cytoplasm to get the individual cells segregated from each other. Implementation of the methods and additional information are available online https://sites.google.com/site/cellsegmentationhcs/.

### Cell cytoplasm segmentation

The first step in our segmentation methodology is robust cytoplasm/background segmentation. As we mentioned earlier, there are many aberrations linked with high-throughput fluorescent microscopy imaging systems. Briefly described, the images typically have low contrast, with blurred regions around the image corners, varying signal strengths, inhomogeneous cell interiors and they also sometimes have uneven illumination. Generally, cytoplasm images appear to be most affected by these problems as far as their accurate segmentation is concerned.

Apart from these imaging related challenges, the other challenge that we face is posed by our dataset which includes cells with high phenotypic variability. Examples of challenging phenotypes are ruffles and spikes in cell boundary and other kinds of outline variations. A segmentation method robust enough to detect such fine details from the noisy and low contrast images is needed for distinguishing different phenotypes. Our approach is to first apply enhancement and correction to the images before applying any segmentation method. Here, we use a cascade of three image and contrast enhancement filters for image pre-processing and a multi-scale approach for getting the desired initial cytoplasm/background segmentation. Block (A) in Figure 1 shows the steps performed in getting initial cytoplasm segmentation.

#### Image pre-processing

**Figure 2:**
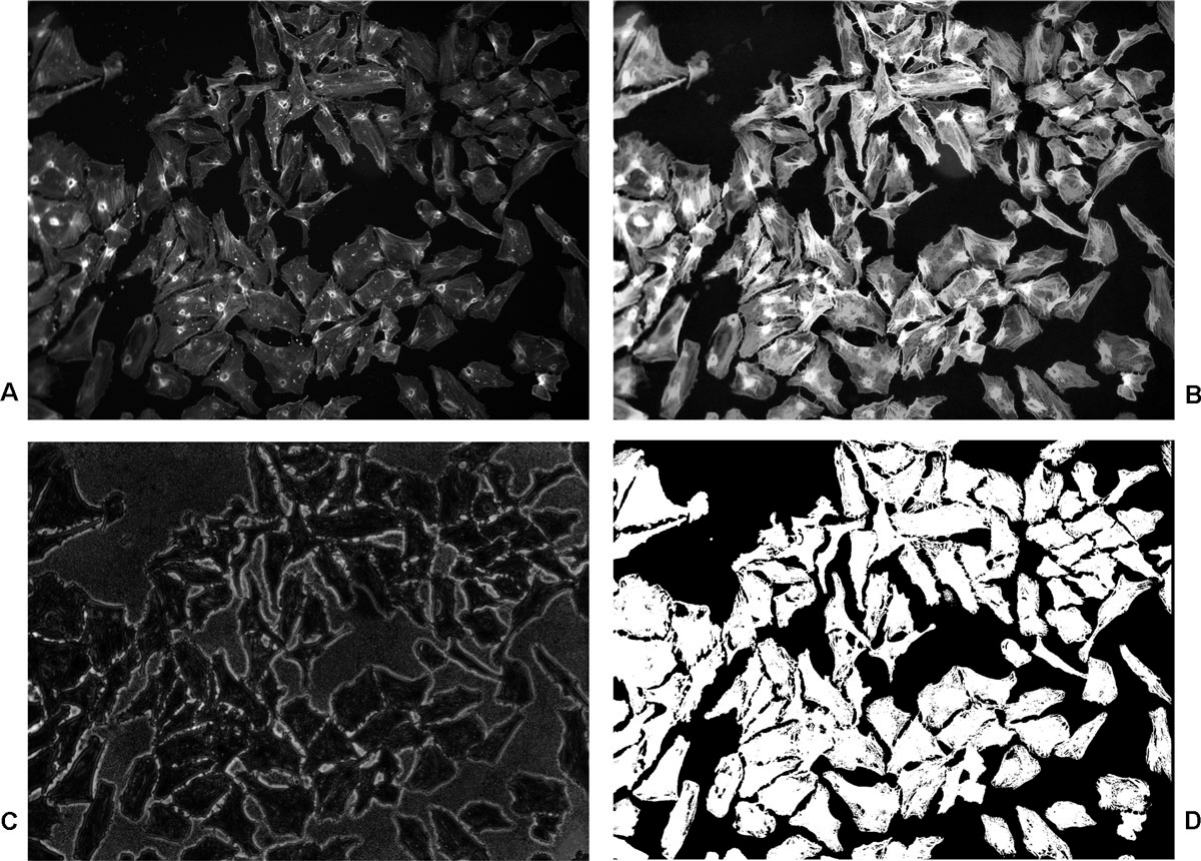
**Image pre-processing and cytoplasm/background segmentation**. Image pre-processing and cytoplasm/background segmentation. (a) An actin-channel cell microscopy image showing the cell cytoplasm and (b) the result of pre-processing. (c) The coefficient of variation image of scale-space representation and (d) the resulting cytoplasm/background segmentation. The size of the image is 1040*×*1392 pixels.

A cascade of image and contrast enhancement filters is used to preprocess the image to solve most of the above mentioned problems. First, contrast-limited adaptive histogram equalization [21] is applied to enhance the contrast of the image. The image is divided into 8*×*8 tiles and contrast of each tile is enhanced and the neighboring output tiles are combined using bilinear interpolation to avoid artifacts. In homogeneous regions of the image, over-saturation is avoided by clipping the high histogram peak occurring due to many pixels with similar intensity values. Then we applied opening by morphological reconstruction to the contrast enhanced image (mask) using a marker image. The marker image is created by eroding the mask image by a flat disc-shaped structuring element of radius of 5 pixels. The advantage of performing opening by reconstruction over conventional morphological opening is that, after opening, the topology of the cytoplasmic regions remains intact. It mainly smoothens out spurious high and low valued pixels and tackles the problem of uneven and varying actin signal. Finally, contrast of the image is adjusted once more by saturating 1% of the high and low intensity valued pixels. We will see that this is also beneficial for the image processing at the next stage. Figure 2(a) shows an original actin channel cytoplasm image and (b) the corresponding pre-processed image.

#### Multi-scale coefficient of variation based cytoplasm segmentation

After pre-processing the cytoplasm image, the initial cytoplasm/background segmentation is performed using our novel approach. Difference of Gaussian is a well-known technique used to enhance the edges in the image, especially the ones corrupted with noise [22]. On the other hand, for a stack of brightfield images, coefficient of variation has been found to be effective in contrast and details enhancement [23]. Our approach effectively combines the characteristics of these two approaches. It is based on coefficient of variation of the multi-scale Gaussian scale-space representation of the cytoplasm images to enhance the low contrast cytoplasmic regions. For an image *f *(*x*, *y*), its Gaussian scale-space representation is a family of derived signals [24] given by

(1) $$L\left(.,\;.;t^{2}\right)=g\left(.,\;.;t^{2}\right)*f\left(.,.\right);\;t\geq 0,$$

where

$$g\left(x,\;y;\;t^{2}\right)=\frac{1}{\left(2\pi t^{2}\right)}e^{-\left(x^{2}+y^{2}\right)/2t^{2}},$$

is a Gaussian kernel of increasing width *t *and ∗ stands for the convolution operation. The parameter *t *is a parameter indicating the scale and at *t *= 0 the scale-space representation is the image *f*(*x*, *y*) itself. For increasing value of *t*, *L *is an increasingly smoothed version of *f*(*x*, *y*) with lesser details in the image. In our study, the scale-space representation is composed of seven images obtained at scales *t *= [0, 1, 2, 3, 4, 5, 6] corresponding to the original image and their coefficient of variation image *f_COV _*is given by

(2) $$f_{COV}\left(x,\;y\right)=\frac{\sqrt{E\left[{\left(L\left(.,\;.;t^{2}\right)-E\left[L\left(.,\;.;t^{2}\right)\right]\right)}^{2}\right]}}{E\left[L\left(.,\;.;t^{2}\right)+\varepsilon \right]},$$

where *E*[*·*] is the expectation operator and *ε *= 1 is used to avoid probable outliers due to division by zero at pixel locations with zero intensity value. This leads to an image with higher values at image background and the cytoplasm outline pixels and relatively lower values for cytoplasmic regions of the image. Moreover, due to the standard deviation of stack of blurred images at different scales, it also enhances the edges and highlights the less bright spikes and ruffles of the cytoplasm. This also helps in differentiating the image background pixels from the less bright regions of the cytoplasm caused by intensity inhomogeneities. Adding the inverse of this image, after normalization, to the image *f*(*x*, *y*) leads to an enhanced image *f_enh_*(*x*, *y*) with cytoplasm pixels clamped at a more higher value while background pixels at a relatively small value, that is,

(3) $$f_{enh}\left(x,\;y\right)=f\left(x,\;y\right)+\left(2^{b}-1-f_{COV}\left(x,\;y\right)\right),$$

where *b *is the number of bits used to represent the image. This enhancement in image increases the difference between the darkest cytoplasm pixel and the brightest background pixel and a simple intensity threshold-based method such as Otsu segmentation [25] is able to give the desired cytoplasm/background segmentation. Figure 2(b) shows a gray-scale pre-processed cytoplasm image, 2(c) the coefficient of variation image and 2(d) the resulting image with cytoplasm/background segmentation. From the figure, it is quite evident that our method is able to detect the cytoplasmic regions correctly despite the presence of intensity inhomogeneities.

### Classification-based cell cytoplasm outline detection

The cytoplasm segmentation obtained in the previous step still has cytoplasms of different cells touching each other. This is the step in which we detect the cytoplasm outlines and apply them to the result of the previous step for getting the whole cell segmentation. As we mentioned earlier, even if the cytoplasm outlines are invisible, especially in the regions where cytoplasms clump, the intensity and other features of the pixels with underlying outlines still closely match the features of outline pixels that are clearly visible. This leads us to an approach in which a classifier is trained to classify a pixel as either outline or non-outline based on the set of local features extracted from the image pixels.

A large set of generic pixel-level features is extracted from the training image using a set of filter banks, see Table 1. Using these features and training labels obtained from manually outlined image(s), a classifier is designed utilizing sparse logistic regression classification framework which has feature selection property inherent to it. For any test image, only the features selected by the classifier are extracted and using the designed classifier the image pixels are classified as outline/non-outline pixels, see block (B) in Figure 1.

**Table 1:** Filtering operations and the filter parameters for computing pixel-level features from training images.

Operation (Feature)	Parameter	Values	Total
Gaussian low pass	kernel width *σ*	3:2:49	24
Integrated pixel intensity	kernel size	3:2:9	04
Laplacian of Gaussian	kernel width *σ*	3:2:49	24
Difference of Gaussian	kernel width *σ*		05
Morphological top-hat	kernel size	3:2:49	24
Morphological bottom-hat	kernel size	3:2:49	24
Local binary pattern	(quantization,		
and contrast	radius)	(8,1)	02
Variance	kernel size	3:2:49	24
Order statistics			
(Min., Med., Max.)	kernel size	3:2:7	09
Haralick (13-features)	kernel size	5:2:15	78
Gabor filter	kernel size,	5:2:15,	
	freq. *f*,	1/4:1/4:3/4,	
	orientation *θ*	0:*π*/4:3*π*/4	72

Total number of features.			290

#### Extraction of features

The complexity and accuracy of a classifier depends upon the number and distinguishing nature of the features used for classifier design. Selection of the most informative features from a list of candidate features reduces the model complexity yet it needs to be performed such that the model yields high classification accuracy. Sparse model using only a subset of the available features allows us to keep the initial feature set large with as many general and redundant features as desired. Moreover, the benefit of using large and general rather than small and problem-specific feature set is that the framework generalizes to other similar classification problems. Hence, we employ an exhaustive set of generic linear and non-linear features knowing our feature selection technique has been successfully used for building sparse classification models in similar use cases [26].

In our study, pixel-level features are extracted from 2D cytoplasm images by applying a large set of filters on them, both in spatial and transform domain, with varying parameters. In [26], the authors use a large generic set of intensity-based features along with textural feature such as local binary pattern (LBP) [27] for image segmentation. Our cytoplasm images possess interesting texture characteristics which might be useful in classification of image pixels. Therefore, in addition to the local binary patterns and other intensity features used in [26], we also incorporate texture features such as the ones obtained from Gabor filters [28] and Haralick [29] features in our classifier design. The feature set comprises general intensity, edge, texture (scale and orientation) based local features which are computed in the pixel neighborhoods using filters with varying kernel sizes. Table 1 lists all the features that are computed for the training images.

#### Design of classifier incorporating feature selection

High-dimensionality of the observations leads to the risk of over-fitting at the cost of generalization of the solution and reduction of feature space is desired. However, selection of the most informative features from a feature set for modeling data characteristics has always been problematic. In case of multiple linear regression modeling, regularization is a process which adds a penalty term to the least square prediction error to shrink the magnitude of model coefficients towards zero. Thus a sparse solution with only few non-zero coefficients is obtained and feature selection is performed automatically. Least absolute shrinkage and selection operator (LASSO) [30] is a technique which penalizes the error function using *l*_1_-norm of coefficient vector along with a regularization parameter *λ >*0 which controls the sparsity of the solutions. This is another characteristic of this framework, that is, its provision of a set of solutions which usually has increasing sparsity for an increasing value of *λ*. The advantage in it is that it helps in choosing a solution with as many features desired with little or no major change in the classification result, that is, a solution with a small trade-off between accuracy and model sparsity/complexity.

Using such framework, a classifier with sparse model is designed by taking the advantage of logistic function to describe the class probability *p*(*o_i_|***x***_i_*) of pixel *i *belonging to outline by

(4) $$p\left(o_{i}|x_{i}\right)=\frac{1}{1+e^{\left({\text{β}}_{0}+{\text{x}}_{i}^{T}\beta \right)}},$$

where *o_i _*represent the class "outline" and probability for class "non-outline" *n_i _*is given by *p*(*n_i_|***x***_i_*) = 1 - *p*(*o_i_|***x***_i_*), $x_{i}\in {\mathbb{R}}^{p}$ denotes the feature vector of the *i^th ^*pixel and (*β*_0_, ***β***) is the coefficient vector which is estimated by maximizing the penalized log-likelihood given by

(5) $$\overset{N}{\underset{i=1}{\sum }}\left\{\text{log}p\left(o_{i}|x_{i}\right)+\text{log}\left(1-p\left(o_{i}|x_{i}\right)\right)\right\}-\lambda ∥\beta {∥}_{1},$$

whose quadratic approximation gives rise to an equivalent penalized iteratively re-weighted least squares problem that can be solved by coordinate descent algorithm [31].

#### Training and classification

In order to perform training and classification, manually created benchmark images with cytoplasm outlines are used. We have a set of training samples, around 550 cells (5 images) and 1250 cells (16 images) for *Test Case I *and *Test Case II*, respectively, segmented manually by expert biologists, see details regarding image acquisition in later section. It is worth-mentioning that, while choosing the images for benchmarking, the criteria was to pick those images which contain most of the image area covered with cells and also the chosen images present one of those cases which are the most challenging as far as getting accurate segmentation is concerned. Since all the images are 1040*×*1392 (*Test Case I *) and 400*×*400 (*Test Case II *) in size, even the pixels of a single image are sufficient enough to train the classifier, especially the classifier of our type which is capable of dealing with even *P *≫ *N *cases. Therefore, one of the images is used solely for training of the classifier while the rest of the images are used for evaluating the classifier. This way we made sure not to use the same data for both training and testing.

For training, 500 positive (outline) and 500 negative (non-outline) samples are picked at random from 1447680 or 160000 samples in the benchmarked image of cytoplasm outlines. For these 1000 samples, all the 290 features listed in Table 1 are extracted from the corresponding cytoplasm image. This training data of 1000*×*290 feature vector along with 1000*×*1 target labels is input to the regularized logistic regression classifier. For testing, only the selected features are calculated for every pixel in the test images to be used with the selected model for outline classification.

In order to estimate the optimal classifier model coefficients, 10-fold cross-validation is performed on the training data to estimate the prediction error of all the solutions obtained for different values of regularization parameter *λ*. The solution which gives the minimum prediction error is generally chosen, however, it can be left to the discretion of the designer to pick an even more sparse solution with little or no major impact on the final classification results. In our case, we observed that models within one standard error of the mean cross-validation error do not change the classifier output significantly. Finally, the selected model for the classifier gives the posterior probability values for the pixels in the test image which is used directly to find the class label (outline/non-outline) for every pixel.

### Post-processing

Post-processing of the classifier outputs is generally a complementary part of any classification framework. One of the techniques used for post processing exploits the contextual information obtained either from the targeted patterns, which, in our case is cytoplasm images, or from some other source related to them. The classifier that we obtained to classify image pixels as outline/non-outline gives accurate yet coarse results. The coarseness mainly comes from the fact that sometimes the pixels interior to the cytoplasm are given the outline labels due to similarity of their features with outline pixels which was actually caused by varying and inhomogeneous actin signal. Moreover, due to binary outputs, that is, the threshold probability value of 0.5, the classifier tends to give thick outlines because many pixels close to the actual outline have similar features with little variations among them. Also, again due to varying signal strength or due to noise, quite often the detected outlines are non-connected, whereas, the desired solution is to have closed contour outlines for cytoplasms. Therefore, we need to refine the classifier output and transform it in such a way that we get single-pixel length closed outline contours.

In eukaryotic cells, nucleus is the main indicator of a cell. We have the DNA-channel nuclei images which provide a solid basis to find the individual cells, or to detect individual cell cytoplasm outlines in the actin-channel cytoplasm images. In cell images, nucleus is generally located at the central portion of the cell. Most importantly, we can certainly assume that the pixels occupied by the nucleus can never be occupied by the cell outlines. Therefore, nuclei images provide contextual information for post-processing of the classifier output. Mainly, they are used to filter out the misclassified outline pixels lying inside the cell. In the same context, they are also used to refine the result of initial segmentation to fill underlying small holes occurring due to intensity inhomogeneities. This image is then inverted and unified with the filtered outline image to further strengthen the outlines.

Once the outlines are filtered, their thinning is performed by morphological skeletonization to get single-pixel length outline contour. Skeletonization is preferred over morphological thinning since it gives not only accurate contour in terms of its location but it also gives non-connected branches wherever available. These branches occur either due to discontinuous outlines or due to some noisy structures in the original cytoplasm images, and help in getting closed contour outlines. Decision on whether to join these non-connected branches or not is taken on the basis of object correspondence at the nuclei and cytoplasm level. In order to find the correspondence, the thinned outlines are applied on the initially segmented images to get the first-stage cytoplasm segmentation. Due to false positives and false negatives in the outlines classification we get over- and under-segmentation. To deal with this, nuclei images are used to perform an additional step of splitting and merging.

In the splitting and merging step, firstly, nuclei image is used to morphologically reconstruct the first-stage cytoplasm segmentation image. This separates objects or cytoplasmic regions with and without a corresponding nucleus. The latter ones are saved to be merged in a later part of this step. In the former case, we have two types of correspondences: one-to-one correspondence between cytoplasm and nucleus and one-to-many correspondence between cytoplasm and nuclei. In the former case, there is one nucleus for every cytoplasm which is often the case in our images as there are very few multinuclear cell phenotypes. Morphological closing is applied to such objects to smoothen inside of cytoplasm and to remove any non-connected branches occurring due to noise or intensity inhomogeneities.

In the case of one-to-many correspondence, the respective non-connected branches in outline are extracted and dilated to close in the gaps. Skeletonization and morphological reconstruction are applied again to split the regions into nucleus-bearing regions and non-nucleus-bearing regions. It is worth-mentioning that no extra splitting approach is used in order to get one cytoplasmic region per nucleic region. The reason is that the nuclei used for finding correspondence are themselves found to be affected by over-splitting and an attempt to forcefully split a cytoplasmic region despite the absence of outline would result in cytoplasm over-segmentation translated from nuclei over-segmentation. Moreover, our approach also helps in retaining the morphology of the multinuclear cell phenotypes.

**Figure 3:**
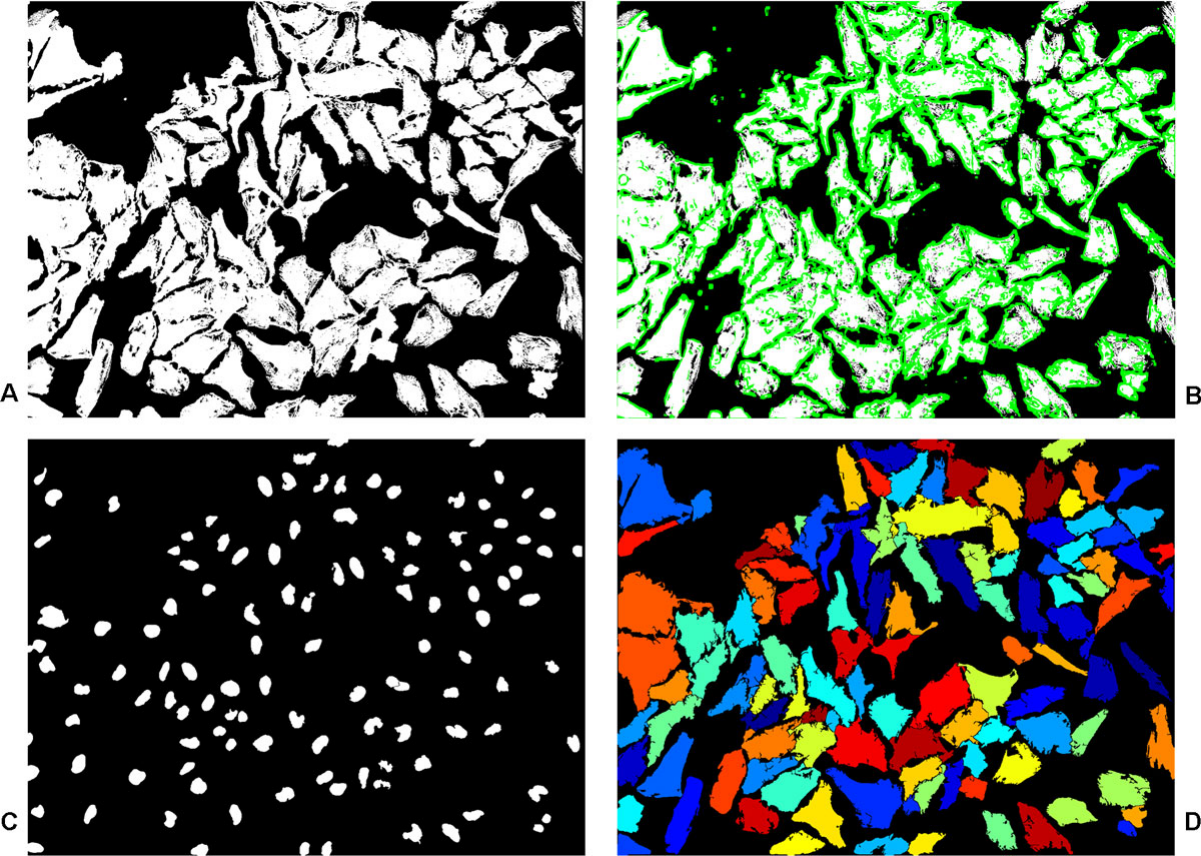
**Outline detection and post-processing**. Outline detection and post-processing. (a) An image after initial segmentation. (b) Resulting outlines (green) from classification of image pixels into outline/non-outline pixels. (c) Corresponding DNA-channel nuclei image, segmentation obtained from method in [6]. (d) Final segmented image after post-processing. The size of the image is 1040*×*1392 pixels.

Finally, region merging is performed to merge all the non-nucleus-bearing regions resulting from the previous step with the separated nucleus-bearing cytoplasmic regions. Candidates for merging are obtained by dilating the to-be-merged regions and finding the overlapping regions in the nucleus-bearing cytoplasmic regions. Since the cells in our image set are mostly convex, therefore, in the case of more than one candidates, the one which gives the largest solidity is chosen. The process is repeated for a couple of more iterations so that regions that do not have an overlapping cytoplasm initially, due to being away from a cytoplasmic region, may have one now due to their adjacent regions being merged with a cytoplasmic region in the previous iteration. In the end, morphological operations are performed to remove h-connectivity as well as 8-connectivity of the objects and to fill small holes in them. Block (C) in Figure 1 outlines the steps performed in post-processing to get the final segmentation result. Figure 3 shows the results of outline detection and post-processing for the segmented image of Figure 2.

## Results and discussion

To study and analyze the performance of our segmentation methodology, we test it against two challenging test cases. Both of them consist of image sets of different cell types with cells of varying size, shape, texture and degree of overlap. The first case is challenging in the sense that it contains images with high cell density with large variation in the shape as well as in size of the cells. The second test case is more of a validation case because it not only contains images from publicly available dataset with ground truth benchmarking, but it also presents an altogether different set of images from the first test case. This enables testing the generalization of our framework. The challenging aspect of the second case, similarly as for the first case, is that the cells are such tightly clustered with virtually no indiscernible boundaries that even accurate manual segmentation is sometimes impossible. Moreover, in both the cases, the extensive variation in signal strength, intensity inhomogeneity and low contrast make the segmentation task even more challenging.

### Image acquisition

The details about the experimental settings to perform image acquisition for compiling the dataset for *Test Case I *and *Test Case II *are given below.

#### Test Case I

Experiments were conducted in a 384-well plate format imaging *HeLa CCL-2 ATCC *cells using Molecular Devices ImageXpress microscopes (10*× *objective; 9 sites per well, Channels DAPI: DNA, GFP: pathogen, RFP: actin) with robotic plate handling. The objective was 10X S Fluor. Image binning was not used. Gain was set to low (Gain1). Laser-based focusing was enabled and image-based focusing was disabled. The dynamic range was set to 12 Bit Range. Z-Offset for Focus was selected manually and *AutoExpose *was used to get a good exposure time. Manual correction of the exposure time was applied to ensure a good dynamic range with low overexposure, when necessary. The size of each image is 1040*×*1392 pixels. Manual benchmark creation was performed by biologists where cell cytoplasm outlines are drawn. Due to the presence of multinuclear phenotypes, there are few cases of multiple nucleus per cytoplasm. Five images containing around 550 cells were taken which were representative of most of the problematic cases not solved well by a widely used method from [32].

#### Test Case II

In this test case we use images of *Drosophila melanogaster Kc167 *cells which were stained for DNA (nuclei) and actin (cytoplasm). "Images were acquired using a motorized Zeiss Axioplan 2 and a Axiocam MRm camera, and are provided courtesy of the laboratory of David Sabatini at the Whitehead Institute for Biomedical Research. First, nuclei were outlined by hand. The nuclear outlines were overlaid on the cell images, and one cell per nucleus was outlined" [33]. There are 16 images in the dataset with size 400*×*400, 450*×*450 and 512*×*512 pixels, containing cells of around 25 pixels in diameter with an average of 80 cells per image. The motivation for using this image set primarily comes from its public availability and benchmarking. Also, these images provide challenging segmentation tasks which have also been worked upon previously, such as in [16,34,35]. This helps in examining the proposed method in comparison to the results obtained from these state-of-the-art methods.

### Segmentation quality metrics

To evaluate the accuracy of our segmentation method and to quantitatively compare it with other methods, we obtained performance metrics at two different levels: pixel level (cytoplasm image) and object level (both nucleus and cytoplasm images). The performance metric that we used is F-measure (*FM*), like we did in [6,36], which is the harmonic mean of Precision (*PR*) and Recall (*RC*) and is given by

(6) $$FM=\frac{2}{\frac{1}{PR}+\frac{1}{RC}},$$

where

(7) $$PR=\frac{TP}{\left(TP+FP\right)}\;\;\text{and}\;RC=\frac{TP}{\left(TP+FN\right)},$$

where *TP*, *FP *and *FN *are true positive, false positive and false negative, respectively, with respect to the benchmarked images. The higher the rate of true values, the lower the rate of false values and the higher would be the segmentation accuracy.

Pixel-level measures give an insight into how accurate the obtained segmentation is, in terms of correspondence between cells in segmented image and benchmarked image. For each cell in the benchmarked image, based on maximum overlap, a corresponding cell was found in the segmented image. *TP*, *FP *and *FN *values were obtained at pixel-level and *FM *value was obtained. In order for correspondence to be true, a threshold value of *FM_th _*= 0.6 was used as it was used in [16]. Once an object correspondence is found, the object was removed from the segmented image and was not considered for any other object in the benchmarked image. In this way, only one-to-one (*TP*), one-to-none (*FN*) or none-to-one (*FP*) correspondence was obtained between the benchmarked image and the segmented image. This also accounted for the object-level measure for cytoplasms, that is, every one-to-one correspondence meant an increase in cell count. Object-level measures for the nuclei were also obtained in a similar way to get the nuclei count.

It is worth-mentioning that while finding correspondence for cytoplasms, the nuclei image was not used at all. The reason is that an over-splitting at nuclei level may not always cause over-splitting at cytoplasm level due to true absence of outline. Therefore, using nuclei for finding correspondence may result in wrong quantitative measures.

### Nuclei segmentation

In both cases, nuclei segmentation was obtained by using our framework presented in [6]. However, in that framework we used graph cut segmentation method from [37] which can be replaced with the initial segmentation method proposed here for cytoplasm segmentation. From the results, it has been observed that although the nuclei segmentation framework with our proposed initial segmentation gives less smoother result than the framework with graph cut segmentation but when compared quantitatively it was able to reduce twice as many false negatives as it increases false positives. The reason is that our initial segmentation method was found to be better in detecting objects in low contrast with varying signal strength than graph cut method, even though the applied pre-processing was the same. Although, the final F-measure value was almost similar in either case, the decrease in false negative meant an increase in cytoplasm detection, whereas, a false positive might not be as costly since nuclei image is not affecting the splitting of cytoplasm regions as long as there is no underlying outline detected. For *Test Case II*, we replaced the graph cut-based initial segmentation of the framework in [6] with the initial segmentation method proposed here. As the magnification of these images is different from our images, that is, they have lesser pixels per nucleus, the set of values used for scale needs to decrease in order to avoid objects from getting connected due to larger kernel width. Therefore, Gaussian filtering was performed with smaller kernel width. Hence, the scale-space representation was composed of 7 images obtained at scales *t *= [0, 0.5, 1, 1.5, 2, 2.5, 3] corresponding to the original image to get the initial segmentation as described in cell cytoplasm segmentation subsection.

### Implementation details

**Figure 4:**
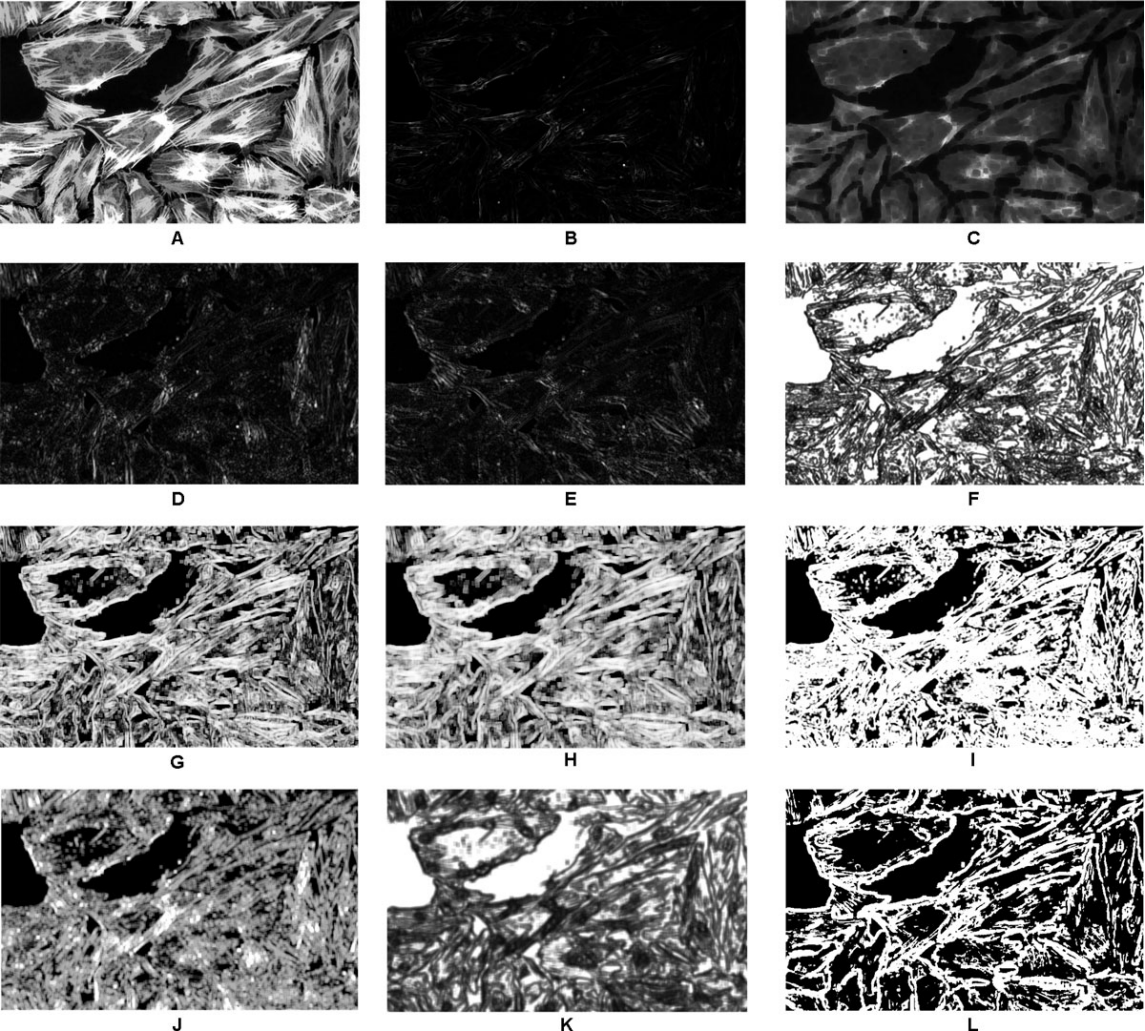
**Visual representation of features used by classifiers**. Visual representation of features used by classifiers. (a) A pre-processed image, (b) *VAR*_3*×*3_, (c) *MIN*_7*×*7_, (d) *f*1*/*4*th*0_5*×*5_, (e) *f*1*/*4*th*3*pi/*4_5*×*5_, (f) *ASM*_5*×*5_, (g) *IMOC*2_7*×*7_, (h) *IMOC*2_9*×*9_, (i) *ENT*_5*×*5_, (j) *DOE*_7*×*7_, (k) *ASM*_9*×*9_, and (l) outlines obtained from thresholding the output of classifier. The size of the images is 700*×*430 pixels.

In this subsection, we describe the procedure and the implementation details of the methodology for obtaining the results. In order to get the quantitative measures for evaluation, we applied our segmentation methodology on the two image sets from the two test cases. First, we obtained nuclei segmentation in the way described in the previous subsection and the values of 600 and 100 were used for allowed minimum area of a nucleus for *Test Case I *and *Test Case II *respectively. Then, cytoplasm/background segmentation was obtained as mentioned in cell cytoplasm segmentation subsection. Finally, the outline/non-outline classifier design gave a sparse model with only eight non-zero coefficients for the *Test Case I *and linear model in denominator of Equation 4 turned out to be

(8) $$\begin{array}{c} {\text{β}}_{0}+x_{i}^{T}\beta =0.2415-44.998*f_{1}+0.010*f_{2}-0.006*f_{3}\\ \;\;\;\;\;\;\;\;\;\;\;\;\;\;\;\;\;\;-0.009*f_{4}+0.068*f_{5}-0.207*f_{6}-0.544*f_{7} \end{array}$$

where *f*_1 _= *VAR*_3*×*3 _stands for variance, *f*_2 _= *MIN*_7*×*7 _for minimum, *f*_3 _= *f*1*/*4*th*0_5*×*5_, *f*_4 _= *f*1*/*4*th*3*pi/*4_5*×*5 _for Gabor filtering frequency and orientation, *f*_5 _= *ASM*_5*×*5 _for angularSecondMoment, *f*_6 _= *IMOC*2_7*×*7 _and *f*_7 _= *IMOC*2_9*×*9 _for informationMeasureOfCorrelation2, see [29] for details. The subscript *x × y *stands for the respective kernel sizes. On the other hand, for the *Test Case II*, the classifier design gave a sparse model with only six non-zero coefficients and linear model in denominator of Equation 4 turned out to be

(9) $$\begin{array}{c} {\text{β}}_{0}+x_{i}^{T}\beta =1.120-0.0165*f_{1}-0.1790*f_{2}-0.360*f_{3}\\ \;-1.54*f_{4}+0.1908*f_{5} \end{array}$$

where *f*_1 _= *ENT*_5*×*5 _stands for entropy, *f*_2 _= *DOE*_7*×*7 _for differenceOfEntropy, *f*_3 _= *IMOC*2_7*×*7 _and *f*_4 _= *IMOC*2_9*×*9 _for informationMeasureOfCorrelation2 and *f*_5 _= *ASM*_9*×*9 _for angularSecondMoment, see [29] for details. Again, the subscript *x × y *stands for the respective kernel sizes. Then, for each of the test images, feature vector of size 1447680*×*7 for *Test Case I *and 160000*×*5 for *Test Case II *were calculated and input to the above models to get the class probabilities using Equation 4. The probabilities were thresholded with threshold value of 0.5 to get outline/non-outline pixels. Finally, post-processing step was performed to get the segmentation done. Figure 4 presents a visual representation of the features used by classifiers given in (8) and (9).

### Results and discussion

Quantitative values from the resulting images were obtained as described earlier in this section and are given in Table 2 and Table 3 for *Test Case I *and *Test Case II *respectively.

**Table 2:** Quantitative values obtained from nuclei and cytoplasm segmentation for Test Case I (See text for abbreviations).

Level (Method)	TP	FP	FN	PR	RC	FM
Nuclei ([6])	458	11	10	0.97	0.97	0.97
Nuclei (CP [32])	466	47	2	0.91	0.99	0.95

Cytoplasm (proposed)	424	23	42	0.95	0.91	0.93
Cytoplasm (CP [32])	409	103	57	0.80	0.88	0.84

**Table 3:** Quantitative values obtained from our segmentation method for Test Case II (See text for abbreviations).

Level (Method)	TP	FP	FN	PR	RC	FM
Nuclei ([6])	76	4	3	0.95	0.96	0.96
Cytoplasm (proposed)	70	9	9	0.89	0.89	0.89

For the *Test Case I*, we have nuclei and cytoplasm segmentation results obtained from CellProfiler 1.0 (CP) implementation [32]. Table 2 also lists the values obtained from them. As we discussed about nuclei segmentation in [6], CP gives low value for *FN *, but at the expense of high value for *FP *. This high value of *FP *at nuclei level got translated into an even higher value at the cytoplasm level. This is because cytoplasm segmentation was purely based on nuclei segmentation and, effectively, one cytoplasmic region was found for every nucleic region. This difference in values for nuclei and cytoplasm segmentation is more due to *FM_th _*value of 0.6 for cytoplasm detection. Since every over-splitting at nuclei level leads to over-splitting of cytoplasm which, most of the time, disqualifies all the cytoplasmic regions corresponding to an over-split nucleus. This is also evident from Table 2 that *FP *for cytoplasm became almost twice of *FP *for nuclei and those extra *FP *also affect the *FN *directly. Finally, the value of *FM *for CP cytoplasm segmentation came out to be 0.84.

As we mentioned earlier, our proposed cytoplasm segmentation mainly needs a low *FN *for nuclei segmentation because, due to cytoplasm-nuclei correspondence-based segmentation, cytoplasms for which nuclei are not detected are merged with other cytoplasms. Although, the *FM *values for CP implementation and our nuclei segmentation do not differ much, the detection error *FP *+ *FN *for our method was 21, which is less than half as compared to 49 for CP implementation.

**Figure 5:**
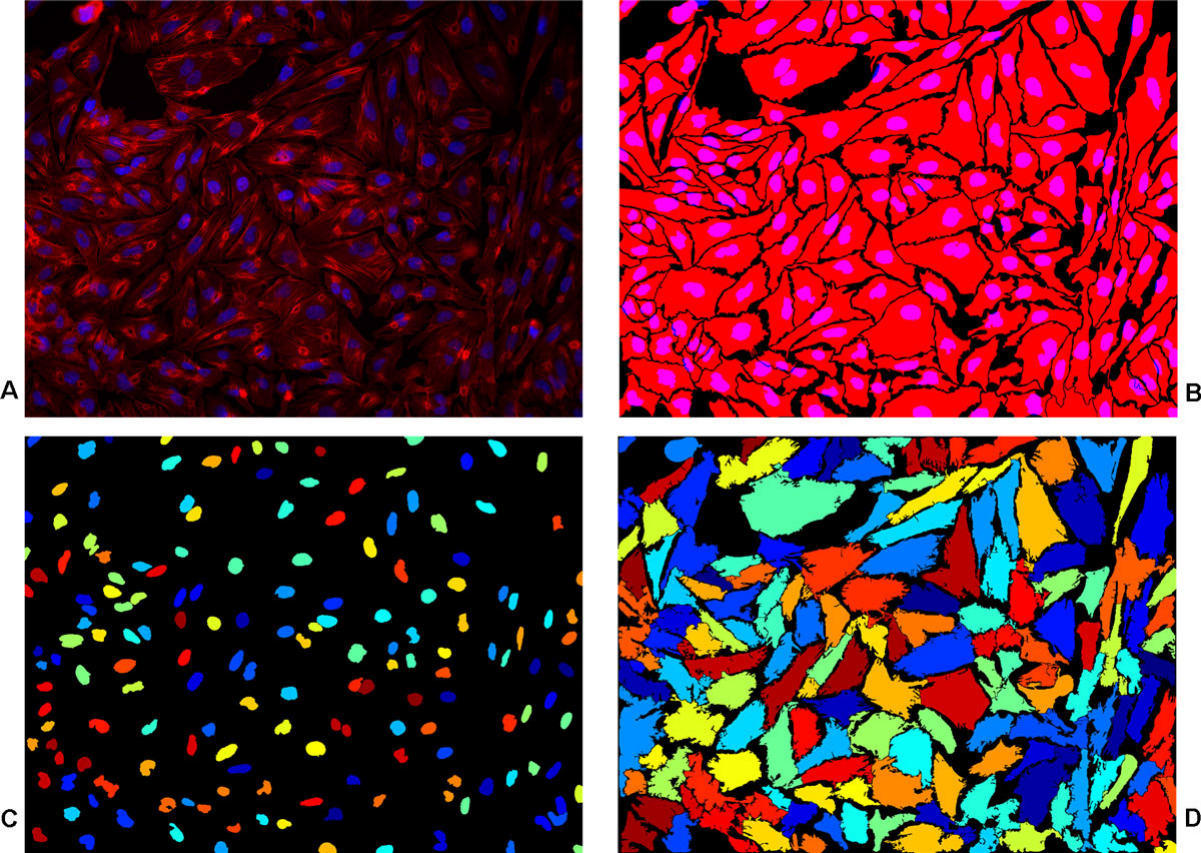
**Cell cytoplasm segmentation for Test Case I**. Cell cytoplasm segmentation for *Test Case I*. (a) A merged cytoplasm (Red)/nuclei (Blue) channel image, (b) benchmark segmentation from biologists, (c) nuclei segmentation from [6] and (d) the result of proposed segmentation. The size of the image is 1040*×*1392 pixels.

In the light of the discussion in the previous paragraph, forced splitting for obtaining one cytoplasm per every detected nuclei did not seem beneficial. However, the *FP *for our cytoplasm segmentation was still found to be twice as much as for nuclei segmentation. The reason is that objects that do not get split into constituent cells were no longer able to correspond to even a single object in benchmarked image because of the constraint of *FM_th_*. Moreover, the consequence of avoiding forced splitting was an increased value for *FN *as some clumped cells did not get detected. A worth-mentioning point is that since the value of *FP *for our nuclei segmentation was low, forced splitting might still have resulted in a similar value of *FP *that we obtained without doing so, but that would have given a much lower value for *FN*. However, the main reason behind not using forced splitting was that we want to retain multinuclear cell phenotypes. The overall segmentation from the proposed method confirms that it outperforms the method from CP with a 9% increase in *FM *value. Another measure that we obtained is the mean value of *FM *for all the correctly detected cytoplasms and it was 0.85 for the proposed method against 0.81 for CP implementation. This also shows how well the cytoplasms correspond among the benchmarked images and our segmented images. Figure 5 presents the segmentation results from the proposed method for qualitative evaluation.

**Figure 6:**
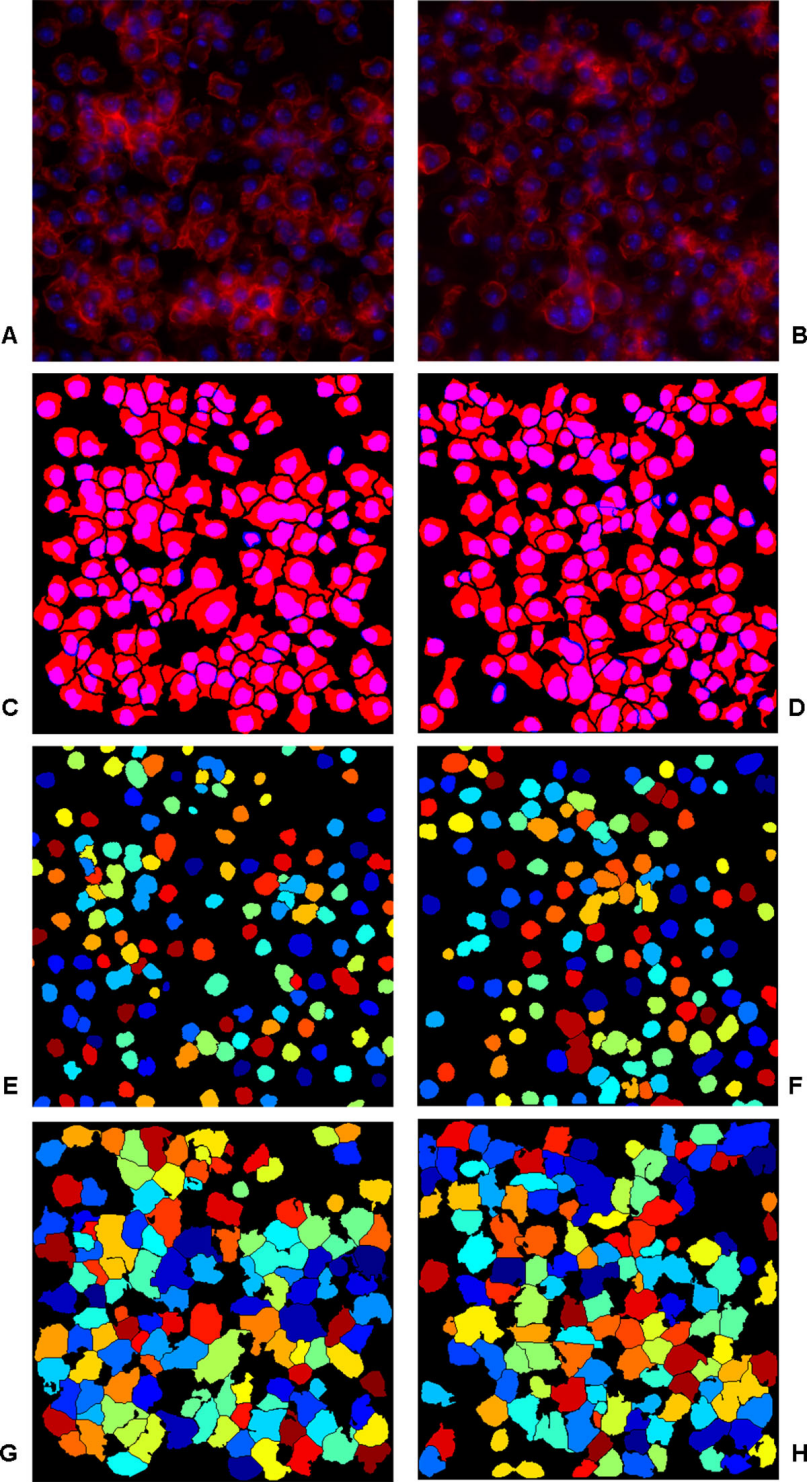
**Cell cytoplasm segmentation for Test Case II**. Cell cytoplasm segmentation for *Test Case II*. (a)-(b) Two merged cytoplasm (Red)/nuclei (Blue) channel images, (c)-(d) benchmark segmentation, (e)-(f) nuclei segmentation from [6] and (g)-(h) the results of proposed segmentation. The size of the images is 450*×*450 pixels.

The same images of *Test Case II *were used for performance evaluation of the cell nuclei and cytoplasm joint segmentation presented in [16]. Comparing the given values of *TP*, *FP*, and *FN *with our obtained values for cytoplasm segmentation, it can be said that we got similar or slightly improved results. However, it is difficult to say whether the difference has any significance. Moreover, the *FM *value from our method for nuclei detection is 0.95 as compared to the *FM *value of 0.80 reported in [16]. This suggests that our method outperforms a recently proposed method which was also reported to be computationally quite expensive. Figure 6 shows the results of the proposed method for two images from *Test Case II*.

Finally, it is evident from the obtained qualitative as well as quantitative results for both the test cases that the proposed method was able to produce accurate results, see Table 2, 3 and Figure 5, Figure 6. Moreover, considering that both the test cases provide completely different set of images with different challenges, the obtained results also demonstrate the generic nature of our framework. In the end, it is worth-mentioning that even though the method uses manually outlined images for training the classifier, it does not depend on user-defined parameters for segmentation.

## Conclusions

In this article we present a novel approach for cell segmentation. The proposed method uses a new combination of pre-processing methods for enhancing the contrast of cell cytoplasm and especially their boundaries by applying coefficient of variation for a multi-scale Gaussian representation of the input image. The enhanced image is used as a basis of feature extraction process, where filtering, texture operations and other generic descriptors are applied for building a large set of features to be used for building a classifier model for cell outline detection. By applying the logistic regression classifier, known to produce sparse models where only a subset of the initial features are used, a rather simple model with a small set of features is obtained, making the classification process computationally feasible. Finally, in post-processing phase, cell nuclei segmentation is used to aid the construction of final cell outlines from the classification output.

In order to validate the segmentation method, we used two image sets with different characteristics. The quantitative results confirm that the method performs consistently for the two datasets and when compared to a widely used method and values presented in literature, it can be concluded that our results are very promising; either improving or matching the results of earlier presented methods.

In conclusion, we expect that learning based methods may be useful in challenging segmentation tasks, such as in high content screening where low contrast cells should be accurately segmented in order to maintain high accuracy among challenging phenotypes. The labeled training samples, in this context: manually outlined cells in a set of images, is a fundamental requirement for using a supervised segmentation method. In high content screening the amount of image data is huge and since also the validation is in most cases done against manually segmented images, we feel that the gain in performance should justify the task of creating the training data.

## Competing interests

The authors declare that they have no competing interests.

## Authors' contributions

Muhammad Farhan carried out the study, developed and implemented the methodology and wrote the manuscript. Pekka Ruusuvuori conceived of the study, coordinated algorithm design and computational experiments, and revised the manuscript. Mario Emmenlauer and Pauli Rämö participated in the design of the study and revised the manuscript. Christoph Dehio and Olli Yli-Harja participated in design of the study and coordination. All authors read and approved the final manuscript.

## Declarations

The funding for publication of the article comes from the aforementioned projects.

This article has been published as part of BMC Bioinformatics Volume 14 Supplement 10, 2013: Selected articles from the 10th International Workshop on Computational Systems Biology (WCSB) 2013: Bioinformatics. The full contents of the supplement are available online at http://www.biomedcentral.com/bmcbioinformatics/supplements/14/S10
